# A Novel Minimally Invasive Reduction Technique by Balloon and Distractor for Intra-Articular Calcaneal Fractures: A Report of 2 Cases

**DOI:** 10.1155/2018/7909184

**Published:** 2018-04-26

**Authors:** M. Prod'homme, S. Pour Jafar, P. Zogakis, P. Stutz

**Affiliations:** Orthopedic Surgery Department, Riviera-Chablais Hospital, Montreux, Switzerland

## Abstract

Treatment of displaced intra-articular fractures of the calcaneus remains a challenge for the orthopaedic surgeon. Conservative therapy is known to produce functional impairment. Surgical approach is plagued by soft-tissue complications and insufficient fracture reduction. We describe a minimally invasive technique that will hopefully improve these issues. We want to present our first experience through two cases. The first was a 46-year-old man who presented with a Sanders type IIBC calcaneal fracture, and the second was a 86-year-old woman with a type IIIBC calcaneal fracture. We introduced 2 Schanz screws in the talus and the calcaneus. After distraction, we introduced an inflatable balloon inside the calcaneus. By inflating the balloon, the articular surface was reduced by lifting it up. Then bone cement was injected in order to maintain the reduction. Additional screw fixation was used in the young patient. Postoperative imaging showed good congruence of the subtalar joint without leakage of cement, for the two cases. After 2 months, the patients had no pain and were without soft-tissue complications. We advocate this technique to perform a minimally invasive reduction and fixation of intra-articular calcaneal fractures because it preserves soft-tissues and provides good clinical results with early weight-bearing.

## 1. Introduction

Calcaneal fractures account for approximately 2% of all fractures, with displaced intra-articular fractures between 60 and 75% of these injuries [1].

Several treatment options are suggested: conservative, including cast immobilisation and/or Harris' traction; surgical, including percutaneous pinning, open reduction and internal fixation, primary arthrodesis and external fixation [2]. The question of surgical versus nonoperative treatment in calcaneal fractures is still controversial with no obvious benefit for some authors [3]. The main surgical difficulties are the reduction of the fracture and the approach. Especially with open approaches, soft-tissue damage is a well-known issue [4].

The calcaneus is continuously subjected to compressive forces with the articular facets of the midfoot and the subtalar joint. Fractures in this area lead to limitation in everyday activities and work [2, 4]. The fees for accident insurance, such as the Suva (Schweizerische Unfallversicherungsanstalt) in Switzerland [5], are substantial: the treatment fees in Swiss Franc were a mean of 19,210 (range 581–49,843). The insurance costs were a mean of 60,656 (range 1759–159,539). The Suva indemnified a mean of 182 days off.

Many authors recommend minimally invasive procedure with good results, supported by studies comparing open fixation and minimally invasive treatment [6] with excellent American Orthopaedic Foot and Ankle Society (AOFAS) scale results [7, 8]. Using Schanz pins and Kirschner wires, cannulated screws, arthroscopically guided percutaneous fixation, and application of bone substitute, with lower complication rates found, the procedures were considered as promising [9].

We present two cases of intra-articular calcaneal fractures, treated by an innovative minimally invasive technique using the combination of a talocalcaneal distractor and a balloon to perform the reduction of the fracture.

## 2. Cases Presentation

### 2.1. Case 1

A 46-year-old man fell from a stepladder corresponding to a height of 1.5 meters hitting his right heel. There was no torsion of his ankle. He presented with heel pain, increased by motion. We hospitalized him in our Orthopedic Surgery Department in order to investigate his lesions and perform the surgical procedure.

Clinically, he presented with a total functional impotence of his right foot. There was a lateral ankle oedema but no trophic complication. Passive mobilization of his ankle was painful, same as the palpation on the external collateral ligament and the Chopart joint. The Lisfranc joint was painless, and there was no laxity. There was no neurologic or vascular impairment.

**Figure 1:**
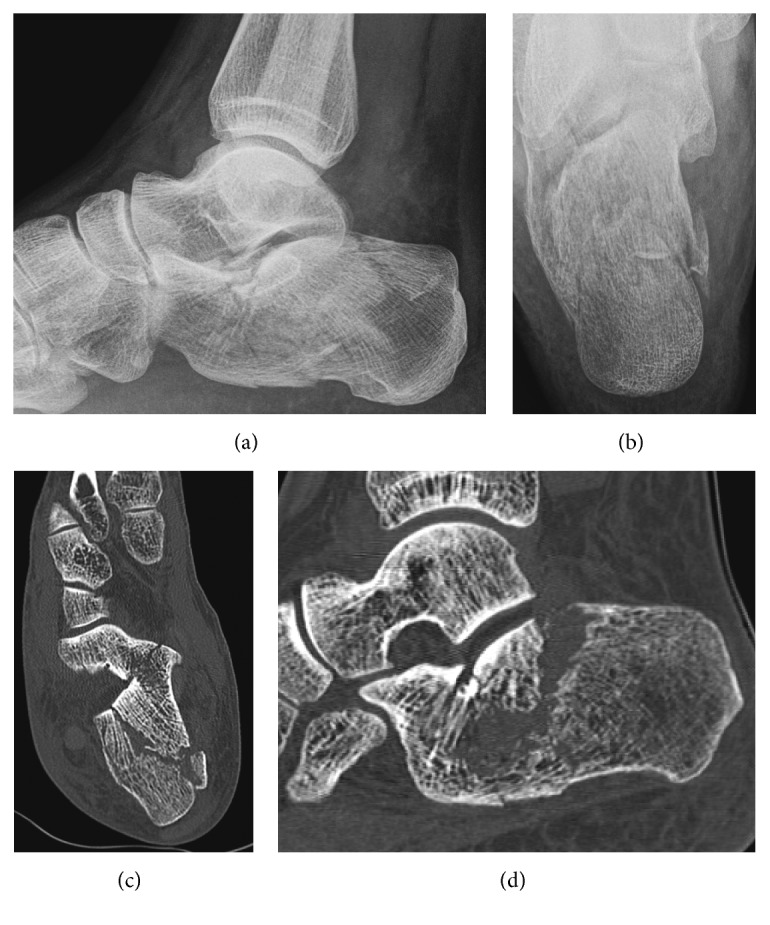
Preoperative imaging of the left foot. The upper part revealed a multifragmentary fracture of the calcaneus, with a Boehler's angle of 5 degrees. The CT scan permitted to classify the fracture as a Sanders type IIBC.

The X-ray of the foot showed an intra-articular calcaneal fracture with a Boehler's angle of 5 degrees, as we can see in Figure 1. The contralateral Boehler's angle was measured as 26 degrees on another X-ray. The CT scan (Figure 1) revealed a multifragmentary fracture of the calcaneus, with a depression of the articular surface. There was also an impingement of the articular surface, on the internal and posterior part. There was no injury of the cuboidocalcaneal articular surface. According to Sanders [4], we classified the fracture as a type IIBC.

#### 2.1.1. Surgical Technique

**Figure 2:**
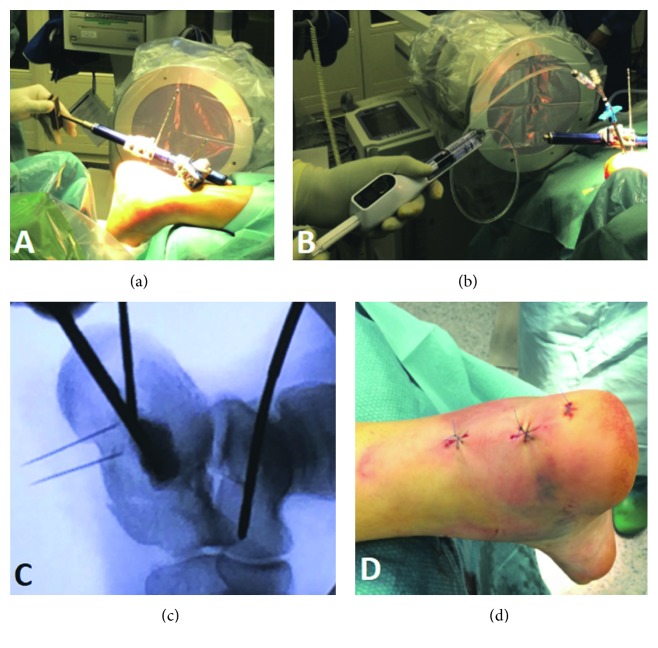
Operative procedure steps: (a) distractor use, (b) balloon reduction, (c) fluoroscopic control, and (d) skin incisions at the end of surgery.

After few days of bed rest, surgery was performed in a ventral position and under general anesthesia. Cefazolin was used for antibiotic prophylaxis. No tourniquet was applied. Under control by image intensifier and by way of stab incisions, we introduced two 4 mm Schanz screws from the posterior side into the talus and the calcaneus. The center of the achilles tendon was longitudinally split before introducing the Schanz screw. Using the distraction device of an external fixator (Hoffmann, Stryker®), we distracted the subtalar joint by counterbalancing the forces of the sural triceps. This also produces a rotation in extension of the posterior fragment and thus a preliminary reduction. By way of a third stab incision, the tip of a Jamshidi needle (Medtronic®) was placed beneath the impacted anterior aspect of the posterior talar joint surface. The needle was replaced by a trocar whose end has the form of a halfpipe. A balloon (Inflate FX™ Gen II Kit, Medtronic) was then introduced into the trocar. By inflating the balloon with Iopamiro® until 400 psi, the depressed joint surface was lifted up and thus disimpacted, respectively, and anatomically reduced. The balloon was then deflated and removed. The cavity which was produced by the balloon was filled with about 4 to 7 cc absorbable phosphocalcic cement (injectable bone void filler; Kyphon®, Medtronic Spine LLC®) in the younger patient or PMMA in the older lady. In the young patient, a screw (diameter 4.0 mm, Synthes®) was introduced such as pointing the cuboidocalcaneal joint. Skin closure was performed by using standard suture. Both patients were operated by the senior author. The main steps are represented in Figure 2.

#### 2.1.2. Follow-Up

We reported no intraoperative nor postoperative complication. The postoperative evolution was uneventful without any soft-tissue problem and with optimal pain control. 15 kg of weight-bearing was recommended since the first postoperative day. For prophylaxis of thromboembolism, Dalteparin natrium was administered daily (5000 IU sc) for the first 10 days and then switched to rivaroxaban (10 mg po per day) for 2 months.

**Figure 3:**
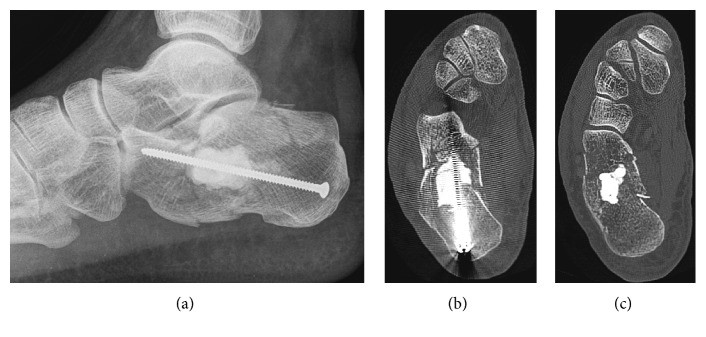
Postoperative imaging, showing a good reduction of the subtalar joint, with a Boehler's angle of 24 degrees, satisfying axis of the calcaneus, and no leakage of cement.

The postoperative imaging of his right foot was in order (Figure 3).

The patient was discharged from the hospital at postoperative day 3.

**Figure 4:**
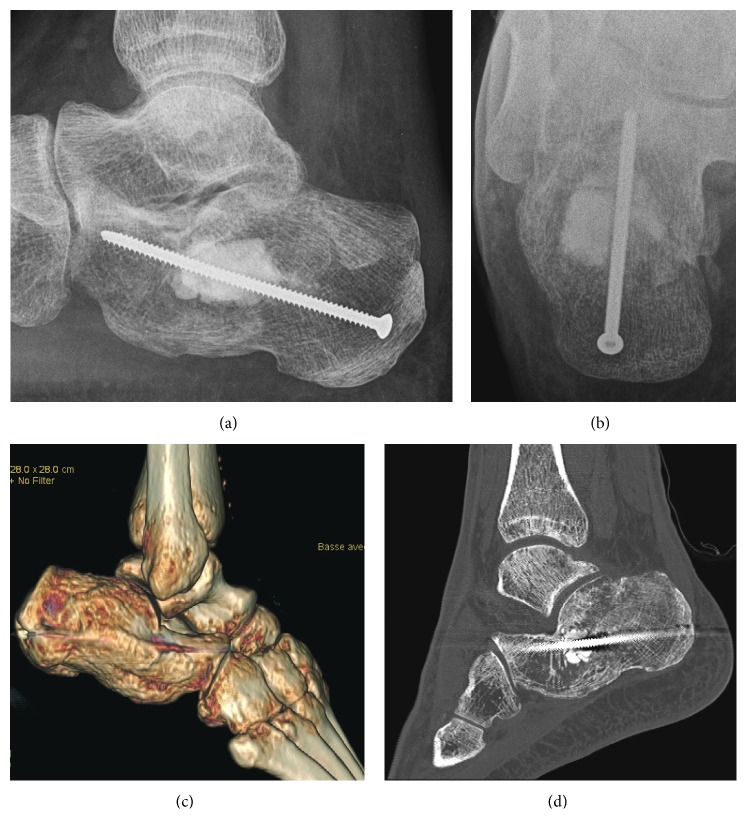
Imaging after 2 months, revealed a partial lack of fracture reduction, but good signs of bone healing.

We reassessed the patient at 2 months. Clinically, he had no pain. The tibiotalar motion was normal, but the talocalcaneal one was 70% of the opposite side. The X-ray revealed a Boehler's angle of 18 degrees versus 24 degrees postoperatively. The CT scan confirmed a partial (25%) lack of the initial reduction (Figure 4).

To prevent from a secondary displacement, we advocated the patient to keep 15 kg weight-bearing on his right foot, during the next two weeks. And after that time, normal weight-bearing was allowed.

After two years, the patient had an AOFAS score of 83/100 which means a good result.

### 2.2. Case 2

Our second patient was an 86-year-old woman with comorbidities, such as active tobacco abuse and osteoporosis, treated by clopidogrel. She fell from her height, describing torsion of her right ankle after walking on a stone. She presented with ankle hematoma and limping with functional impotence of her foot. There was severe pain at the palpation of the lateral malleolus and of the lateral collateral ligament. Ankle and foot motions were limited in all directions. The skin was not impaired.

**Figure 5:**
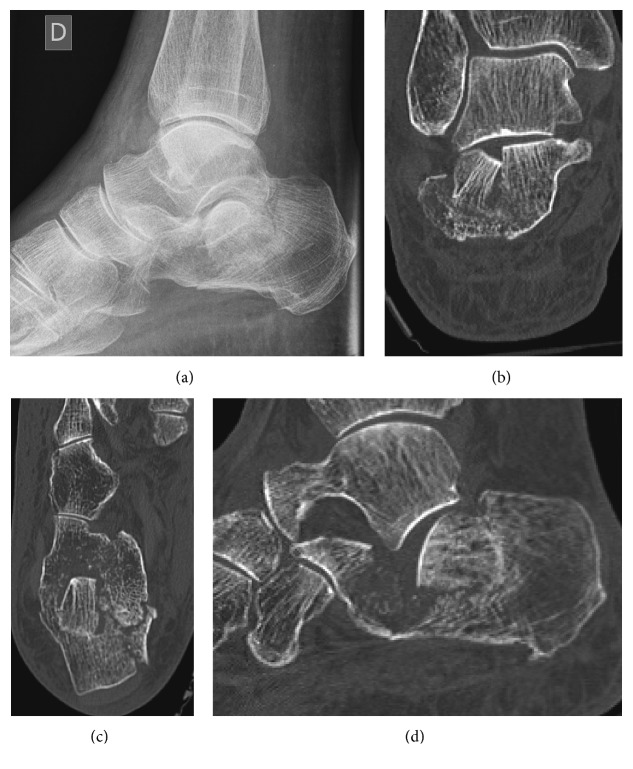
Preoperative radiographs and CT scan of the right foot, confirmed a bony destructive Sanders type IIIBC fracture with a Boehler's angle of less than 5 degrees.

The radiographs of the foot showed a Boehler's angle evaluated less than 5 degrees (Figure 5) and a comminuted fracture of the median and anterior parts of the calcaneus, especially with an intra-articular fracture at the level of the posterior subtalar joint, matching with severe osteoporosis.

The CT scan revealed an osteoporotic bony destructive fracture, more precisely of the average and anterior part of the calcaneus bone. There was a fracture regarding the posterior subtalar joint. We classified it as a Sanders type IIIBC [4].

The surgical approach and the procedure were the same as previously described, except that no screw was inserted and we introduced PMMA cement inside the bone defect, because of severe osteoporosis.

**Figure 6:**
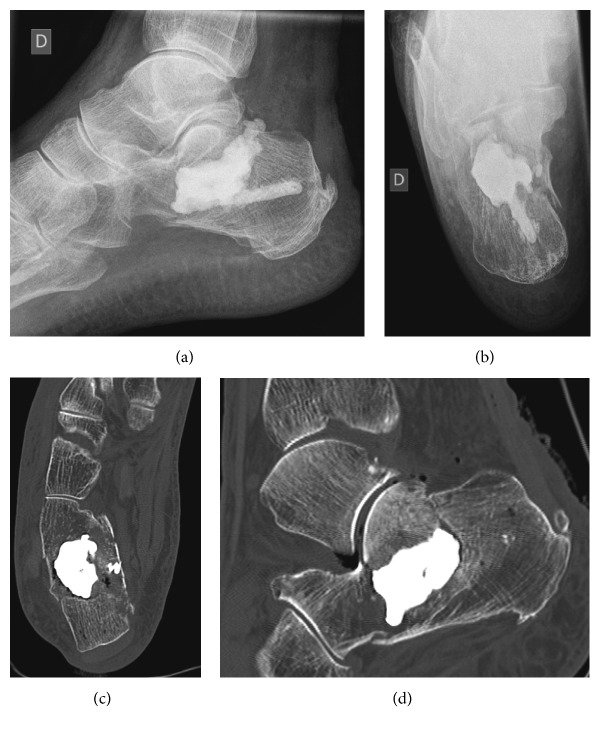
On the upper part, postoperative radiographs showed a Boehler”s angle from 33 degrees. On the lower part, postoperative CT scan showed satisfying alignment of the bone fragments, with presence of a discrete diastasis at the level of the posterior subtalar fracture, but without irregularity of the articular surface.

Postoperative imagings (Figure 6) revealed a satisfying alignment of the bone fragments, with a Boehler's angle at 33 degrees (contralateral side was measured at 36 degrees) and a regular subtalar surface.

The stab incisions presented no complication. The patient could bear full weight-bearing on her right foot with the protection of a simple ankle and foot splint for a 2-month duration. The patient was discharged at postoperative day 10 to enter a rehabilitation center.

We assessed the patient at 2 months postoperative. The injured foot showed a good alignment. The tibiotalar joint motion was about 30 to 40% of the contralateral side. She had no pain. Nevertheless, the X-rays showed a Boehler's angle fall from 32 degrees postoperative to 13 degrees (69%). Unfortunately, after two years of follow-up, the patient was not achievable by phone and deceased.

None of the patients required any reoperation. None of them had pain at follow-up, and no joint subsidence nor arthritis was observed.

## 3. Discussion

Nowadays, the still remaining challenges for the orthopaedic surgeon are the approach and reduction of the calcaneal fracture. Using our described technique, we performed a minimally invasive approach, to decrease soft-tissue damage. Postoperatively, the results were good about pain and motion in the two cases. Malawski and Pomianowski had the same results described in a case reported in 2013 [10]. They used a similar technique for the reduction, with a screw into the talus and calcaneus each, but inserted medially. However, they performed the reduction of the anterior facet by additional maneuvers. Clinically, at 6 months after surgery, normal hindfoot alignment was observed as well as normal range of motion of the ankle joint. The X-rays revealed a fracture union with good shape, but the Boehler's angle decreased to 19 degrees (versus 24 postoperative). According to the Creighton-Nebraska, AOFAS, and Maryland Foot Scores, very good results were observed.

The main argument in favour of our technique is the initial reduction. Thanks to the Schanz screws distraction and the balloon, the intra-articular part of the calcaneal fracture was well reduced, even for the bony destructive fracture.

Thanks to our spine surgery experience, we could similarly utilize an inflatable balloon in corporeal vertebral fracture reductions, according to the balloon kyphoplasty (BK) technique, which is a modified vertebroplasty technique. Vertebroplasty is the standard treatment employed for vertebral compression fractures (VCFs) due to osteoporosis. BK is a minimally invasive procedure that aims to relieve pain, restore vertebral height, and correct kyphosis of the spine. During this procedure, an inflatable bone tamp is inserted into the collapsed vertebral body so as to reduce the fracture. We made use of the procedure with the same objectives, adapted to the calcaneal fracture, to perform the surgery. The devices are well known, their advantages and their own issues. The outcomes described by the Ontario Health Technology [11] showed that BK to treat pain associated with VCFs due to osteoporosis was as effective as vertebroplasty at relieving pain, and it restores the height of the affected vertebra and results in lower fracture rates in other vertebrae compared with vertebroplasty and in fewer neurological complications due to cement leakage compared with vertebroplasty. These results were experienced by the retrospective study from Lee et al. [12], which reported a significantly better quality of life in the group treated by kyphoplasty than in the group treated by vertebroplasty, especially regarding mobility, pain, anxiety, and depression.

An in vitro cadaveric specimens' study from Broome and colleagues assessed the balloon-assisted technique in other indications than VCFs [13]. They assessed 6 proximal tibias and 6 distal radii with a fine-cut microcomputed tomography after creating intra-articular depression fractures and treated them by using a balloon-assisted technique, compared with conventional metal tamp. They showed that the inflatable bone tamp was successful in reducing all distal radius fractures without intra-articular complication, such as overreduction or penetration into the joint, and was even superior to the conventional technique when comminution was present at the articular surface, with minimal residual deformity.

The main limit of our technique was the difficulty to maintain the fracture reduction. The 2-month consultation revealed a partial leakage of postoperative Boehler's angle, despite the respect of no weight-bearing on the foot in case 1. This could be explained by the initial comminution of the fracture, which made the stabilization more difficult, and probably a continuous passive traction by the sural triceps. We supposed that the fracture needed a stronger way to stabilize it, compensating the potential lever arm created by the muscles of the calf, evaluated at 2535 N while walking [14]. But the comminution prevented from doing it with additional screws or K-wires. Probably, another choice would have been to put more cement inside the bone defect, until a partial overcorrection. However, this concept was not supported by the outcomes of Persson's study in 2014 which concluded to avoid surgical overcorrection of the Boehler's angle [15].

Balloon kyphoplasty to treat calcaneal fractures was one of the few described methods in the literature (Table 1). The first publishing was made by Bano et al. in 2009 through a single case [16]. They used a Medtronic inflatable balloon and manometer and made use of calcium phosphate cement. As a result, they showed no complication, full weight-bearing after 7 days, bone recovery after 12 months, and a good Maryland Foot Score with a CT scan revealing no loss of the fracture reduction at the 2-year follow-up.

Gupta et al. directed a study [17] which showed, through a 11 case series, that patients have demonstrated good outcomes in pain (Analogic Visual Scale ranging from 0 to 5, at 10 months) and motion without evidence of postoperative complications such as wound dehiscence, infection, or loss of reduction.

Two studies were conducted by Jacquot and colleagues and firstly published in 2011 [18] on 4 first cases. They showed a good clinical result on all patients and no need for further surgery. They had no pain at the 3-year follow-up for two of them and occasional weather-related pain for the two remaining. They all returned to work and life activities without any limitation. They used a minimally cutaneous approach and then reported no soft-tissue complication. They demonstrated that balloon kyphoplasty to treat calcaneal fractures on a limited series of patients without any risk factors of poor outcome is feasible and may be promising. Later, they published their five-year experience study on 10 patients, and among them one was a bilateral, thus totaling 11 calcaneal fractures [19]. They reported a mean AOFAS score of 84.5 (range from 27 to 100), with a mean Boehler's angle of 15.9 degrees, nearby our radiological results. Only one patient had a poor AOFAS score (27) because of subtalar osteoarthritis. Finally, all patients resumed their work activities at the same level than before the occurrence of the fracture.

Mauffrey and colleagues, in 2012, published their balloon reduction technique through one case of a calcaneal fracture classified Sanders type IV [20]. They described the procedure and pitfalls (top door effect, balloon burst, and cement extravasation) that the surgeon needs to consider.

Furthermore, Biggi et al. [21] published about 11 cases of percutaneous calcaneoplasty. They performed a balloon-assisted reduction and used PMMA cement on 9 cases and calcium phosphate cement on 2 cases. They obtained a postoperative mean Boehler angle of 22.97°, with a mean preoperative angle of 9.91°. Their AOFAS scores after an average of 24 months were excellent in six cases, good in four, and fair in one due to subtalar arthritis.

Finally, Vittore et al. published their results about 20 fractures of the os calcis, treated by a balloon-assisted reduction and a calcium phosphate cement fixation, helped by Kirschner wires [22]. The wires were removed at the seventh day after surgery, and then full weight-bearing was allowed. They showed a mean postoperative Boehler's angle of 25.05 degrees (range from 8 to 36). They reported that AOFAS scores ranged as follows: 4 excellent, 7 good, 6 fair and 3 poor. One patient had secondary subtalar arthritis. They concluded that their approach was associated with pain relief at the first postoperative day, early mobilization, and the tricalcium phosphate cement is a resorbable bone substitute that allows a fair bone stock for hindfoot fusion procedure.

More evidence is needed, despite the good clinical results of the technique described as promising by several authors on short series. A prospective trial with a large sample of patients would be required to assess these options. We advocate the use of the Schanz distractor and the balloon-assisted technique to reduce and treat intra-articular calcaneal fractures, because it provides a good reduction, especially of the intra-articular part which is the most important for the future mobility of the hindfoot and recovering of the gait, and it provides a minimally invasive approach, indicator of a low risk of soft-tissue complications. However, the stabilization of the fracture, on radiological results, remains a concern which should be addressed in further refinements of the technique.

## Figures and Tables

**Table 1:** Literature review of balloon calcaneoplasty procedures.

Author, year	Number of cases	Balloon technique	Relevant results
Bano et al., 2009 [16]	1 case	CaP cement	No loss of reduction after 2 years
Gupta et al., 2011 [17]	11 cases	CaSO_4_ cement + graft	Good results on pain and cutaneous complications
Jacquot and Atchabahian, 2011 [18]	4 cases	PMMA cement	Good clinical results after 3 years, displacement < 1 mm
Jacquot et al., 2013 [19]	10 cases	PMMA cement	Mean Boehler angle 15°, 1 subtalar arthritis
Mauffrey et al., 2012 [20]	1 case	Cement + cannulated screw	No complication, good radiological consolidation
Biggi et al., 2013 [21]	11 cases	9 PMMA, 2 CaP cement	Mean Boehler angle 22.97°, 1 subtalar arthritis
Vittore et al., 2014 [22]	20 cases	Ca3P cement + KW 7 days	Mean Boehler angle 25°, 1 subtalar arthritis

CaP = calcium phosphate; CaSO4 = calcium sulfate; PMMA = polymethyl methacrylate; Ca3P = calcium triphosphate; KW = Kirschner wires.
